# Assessing the validity of digital health literacy instrument for secondary school students in Ghana: The polychoric factor analytic approach

**DOI:** 10.3389/fdgth.2022.968806

**Published:** 2022-09-23

**Authors:** Edmond Kwesi Agormedah, Frank Quansah, Francis Ankomah, John Elvis Hagan, Medina Srem-Sai, Richard Samuel Kwadwo Abieraba, James Boadu Frimpong, Thomas Schack

**Affiliations:** ^1^ Department of Business / Social Sciences Education, University of Cape Coast, Cape Coast, Ghana; ^2^ Department of Educational Foundations, University of Education, Winneba, Ghana; ^3^ Department of Education and Psychology, University of Cape Coast, Cape Coast, Ghana; ^4^ Department of Education, SDA College of Education, Koforidua, Ghana; ^5^ Department of Health, Physical Education and Recreation, University of Cape Coast, Cape Coast, Ghana; ^6^ Neurocognition and Action-Biomechanics-Research Group, Faculty of Psychology and Sports Science, Bielefeld University, Bielefeld, Germany; ^7^ Department of Health, Physical Education, Recreation and Sports, University of Education, Winneba, Ghana

**Keywords:** digital health literacy, factor analysis, polychoric, reliability, students, validity

## Abstract

The emergence of the coronavirus pandemic resulted in the heightened need for digital health literacy among the youth of school-going age. Despite the relevance of digital health literacy among the general public (including students), it appears the measurement of digital health literacy is still a challenge among researchers. Recently, Dadackinski and colleagues adapted existing digital health literacy measures to fit the COVID-19 situation. Since this development, the instrument has been widely used with few validation studies with none in Africa and specifically, in Ghana. The purpose of the study was to assess the validity of the digital health literacy instrument (DHLI) for secondary school students in Ghana using the polychoric factor analysis. We sampled 1,392 students from secondary schools in Ghana. The digital health literacy instrument was administered to the respondents, thereof. The study confirmed the four latent structure of the DHLI. Further, sufficient validity evidence was found regarding the construct validity of the DHLI. The findings from the study support the validity of the DHLI and its utility within the Ghanaian context. With the growing need for digital health literacy among younger people globally, the DHLI provides sufficient grounds for scaling them based on their level of literacy. There is a need for the instrument to be adapted and re-validated in Ghana and among different populations to widen its reproducibility.

## Introduction

The COVID-19 pandemic has affected several lives and sectors of the economy, including education. During the period, students experienced huge psychological consequences threatening their health and well-being (1–7). The sudden onset of COVID-19 was accompanied by an “infodemic”, overabundance of valid and invalid health-related information on COVID-19 (8–12). A recent study revealed that the “infodemic” is so prevalent that 82% of the text ratings examined (i.e., 1,856 of the 2,276 reports) were classified as being “false” (13). During the pandemic, the internet and social media platforms have become important sources of health-related information on the disease and its protective behaviours (11, 14, 15). Conversely, this has led to an explosion of unchecked information and the spread of misinformation (8, 16–19). Recent studies have found that adolescents and young adults (including students) are frequent users of these types of digital media (20, 21), however, they found it challenging dealing with the vast amount of COVID-19 related information as a result of difficulties in seeking, discovering, understanding, judging, and utilising reliable COVID-19 related online information (11, 22–24).

The “infodemic” on the internet and social media could affect students' protective behaviours and mental health. It could also imperil the government and health authorities' efforts to manage the pandemic-related setbacks. Accordingly, for students to effectively navigate the complex information to remain healthy and take relevant precautions using the information available, they would need a high level of digital health literacy (DHL). DHL is the ability to seek, discover, understand, critically appraise health information from electronic sources and apply the knowledge gained to addressing or solving a health problem (25–27). DHL reflects the specific degree of skills and abilities necessary to use digital health technology and services (28, 29). DHL incorporates interactivity across web-based platforms including social media (27). Amid the COVID-19 pandemic, DHL has become an indispensable resource in promoting mental health and psychological well-being among students (27, 30). DHL is a key competence to navigating web-based COVID-19–related information and service environments and addressing the challenges of online health information and services (11, 22).

Despite the relevance of DHL among the general public (including students), it appears the measurement of DHL is still a challenge among researchers. In recent years, several instruments have been developed to measure DHL (31). One of the prominent inventories is the eHealth Literacy Scale (eHEALS) (26, 32). However, the eHEALS has several limitations. First, it focuses on measuring the search for and evaluation of online information, but it does not address critical and interactive health literacy. Second, its validity is unclear and does not consider the new tools provided by the internet and technologies (27, 32, 33). Due to continuous changes in media and technologies, extant researchers advocated for a new instrument for DHL that considers a broad spectrum of applications (27, 34). Subsequently, van der Vaart and Drossaert (27) developed the Digital Health Literacy Instrument (DHLI). DHLI aims to incorporate the skills necessary to use the broad spectrum of applications offered by the internet and communication technologies and give valid information about people's actual competence level. The DHLI includes interactivities on the web, so-called “e-Health 2.0 Skills”. DHLI is composed of seven subscales (i.e., operational skills, navigation skills, information searching, evaluating reliability, determining relevance, adding self-generated context, and protecting privacy), each including three items to be answered on a 4-point Likert scale ranging from 1 (very difficult) to 4 (very easy).

Based on this questionnaire, Dadackinski and colleagues proposed the DHLI in relation to COVID-19 (i.e., COVID-HL) (14, 22, 35). Although DHLI was developed during the COVID-19 period, it can be implemented or used in other conditions. Dadackinski and colleagues adapted DHLI in the context of the COVID-19 pandemic from van der Vaart and Drossaert (27). To generate the COVID-19 DHLI, five out of the seven original subscales were included and modified with respect to COVID-19: searching the web for information on COVID-19 (DHLIsearch); adding self-generated content on COVID-19 (DHLIcont); evaluating the reliability of COVID-19-related information (DHLIrely); determining personal relevance of COVID-19-related information (DHLIrelev); and protecting privacy on the internet (DHLIpriv) (14, 22, 35). Each of these dimensions contains three questions. The internal consistencies (Cronbach *α*) of the first four subscales were acceptable to good (0.70 < *α* < 0.83). Due to low reliability, the protecting privacy subscale (*α* = 0.46) was omitted from COVID-HL.

Since the adaptation of COVID-HL from DHLI, researchers have adapted and validated COVID-HL to ascertain its psychometric properties, factor structure and functionality in different jurisdictions such as Portugal (36), Spain (37), Italy (38) and Korea (39). These validation studies confirmed the four-factor structure and demonstrated good validity and reliability of the scale. For example, in Italy, the Italian DHLI showed good psychometric characteristics. However, the protecting privacy subscale was excluded given the criticalities presented in the validation process. CFA confirmed the four-factor structure (i.e., information searching, evaluating reliability, personal relevance, and self-generated content, (38). Also, in Portugal, Martins et al. (36) discovered that a four-factor structure of the instrument (i.e., information searching, self-generated content, evaluating reliability, and personal relevance) was supported by confirmatory factor analysis and had good internal consistencies. Similar findings have been reported in Korea (COVID-HL-K; 39) and in Spanish-speaking countries like Spain, Puerto Rico, and Ecuador (COVID-HLI-S; 37). The authors of the validation studies recommended representative studies to shed light on different target groups and their COVID-19–related DHLI. Aside these validation studies, the scale (COVID-HL) has been used by other researchers to assess the level of DHL among university students without examining its psychometric properties, factor structure and functionality in other geographical locations such as the UK (40), US (41), Germany (22, 24), Portugal (42), Slovenia (11), China (43), Korea (44), Pakistan (45) and Vietnam (15).

Despite several validation studies on DHLI across several western societies, there is no documented study evaluating the reliability, validity and applicability of the instrument in assessing COVID-19 health-related information during the pandemic period in the sub-Saharan African region. Due to the vulnerability of the youth and their widespread use of the internet through diverse web-based tools (e.g., Facebook, Twitter, Youtube, WhatsApp), secondary school students were targeted as the normative reference group for the current study. In Ghana, the educational system is grouped into three parts: 9 years of Basic education (i.e., early childhood education [kindergarten], primary and junior high school), 3 years of secondary education (i.e., senior high school/vocational and technical schools) and 3–4 years tertiary education (i.e., academic university, technical university, colleges of education and nursing training colleges). Besides, most of the previous validation studies used university students (36–39). This makes it difficult to ascertain the utility and applicability of the DHLI among secondary school students. Additionally, the only DHLI validation study which investigated gender differences, revealed that males had higher levels of DHL as compared to their female counterparts (37). Although this study in question was conducted among university students, it provides a prompt for recent studies to investigate the issue of gender invariance which this present study did.

The issue of health literacy (HL) has been a serious concern for stakeholders in Ghana and has recently been shown to be important for improving universal health coverage (UHC) in Ghana (46). HL is associated with health, well-being and quality of life (47, 48). Conversely, there is no nationwide assessment of health literacy. However, some researchers have examined aspects of health literacy among specific groups. For example, using the adapted Health Literacy Knowledge and Experience Survey Instrument (HLKES), Koduah et al. (49) established that health literacy (HL) knowledge was generally low among student nurses and practicing nurses, with student nurses having significantly lower scores than practising nurses. Similarly, low or limited HL was found among university students (50), street youth (i.e., people between ages 12 and 24 years) (51, 52) and the general population (including students) (46, 47) and women living with breast cancer (48). These studies on HL in Ghana mostly adapted European Consortium for Health Literacy Questionnaire (HLQ-EU-16) (47, 50) and HLKES (49). These instruments (i.e., HLQ-EU-16 and HLKES) are widely used to measure HL, however, they present some limitations such as lack of clear psychometric properties, interactive health literacy and digital health information. Accordingly, this calls for a new scale or instrument to measure Ghanaian's DHL since we are in the digital world. Hence, the overall purpose of the study was to assess the validity of the DHLI for secondary school students in Ghana, using the polychoric factor analysis. Three objectives guided the conduct of the study: (i) to identify the factor solution of the DHLI, (ii) to assess the construct validity of the DHLI, and (iii) to evaluate the measurement invariance of the DHLI based on gender. The outcome of this investigation would be valuable in the development of related policies aiming to increase DHL and compliance with the policies meant to control COVID-19. It would also help to plan and prepare effective communication interventions for this sub-population.

## Materials and methods

### Study setting and participants

This study forms part of the multi-national study by the COVID-19 Digital Health Literacy Network (https://covid-hl.orghttps://covid-hl.eu/). The study setting covered secondary school (senior high school) students within the northern zone of Ghana. This area was the primary focus of this research because of the distinct characteristics possessed by the inhabitants. Particularly, students who had not schooled and/or stay within the region for 10 years or over were excluded. Several reports emerging from the northern belt of Ghana have indicated that more than 40 percent of the inhabitants are living in poverty with 8–9 people out of every 10 persons are living below the poverty line according to the Ghana poverty reduction strategy document (53). Additionally, Ofori-Boateng and Bab (53) indicated that the study setting is characterized by low school completion rates, school drop-out, late start of school and other challenges like working and schooling at the same time. It is not surprising that this present study found young adults between 18 and 25 years who were still in secondary school. A report by the World Bank (54) also mentioned that the populace in the northern zone of Ghana has little chance of breaking out of poverty regardless of the kind of employment they engage in.

This study adopted the descriptive cross-sectional survey design to sample one thousand, three hundred and ninety-two (1,392) secondary school students from the Northern regions of Ghana using a multi-stage sampling technique. The simple random technique was first used to select two regions in the northern part of Ghana. Secondly, five schools from each region were sampled using cluster sampling. Then, the individual students in the schools were purposefully recruited based on whether they had resided in the region for more than 10 years. Seven hundred and two (*n =* 702, 50.4%) of the participants were males while six hundred and fifty-four (*n* = 654, 47.0%) were females and thirty-six (*n =* 36, 2.6%) having diverse sexes. The ages of participants ranged from 14 years to 25 years (Mean age = 18.90; SD = 1.95). Through a random procedure, 500 cases out of the 1,392 were used for the first part of the analyses (exploratory factor analysis, EFA) based on the recommendations of Dimitrov (55) who established that a sample of 400 is appropriate, even in instances where the correlation among the items are low. To improve a more accurate estimate, we added 100 cases for the EFA making a total of 500 as earlier indicated (56). The confirmatory factor analysis (CFA) was conducted with 792 cases which were deemed appropriate for four factors considering the low correlations and high power (57, 58).

## Measures

### Digital health literacy instrument (DHLI)

The DHLI which was originally developed by van der Vaart and Drossaert (27) with 7-subscales and 21-items and later adapted by Dadaczynski et al. (14, 22, 35) to the context of COVID-19 was assessed to determine its factor solution, construct validity, and measurement invariance for gender among secondary school students in Ghana. The study made use of the most current validated form of DHLI (38) with 4-subscales with each having 3-items measured on a 4-point Likert type scale where 1 (Very easy), 2 (Easy), 3 (Difficult), and 4 (Very difficult). The 4-subscales include; Information Searching (e.g., “when you search the Internet for information on coronavirus or related topics, how easy or difficult is it for you to find the exact information you are looking for”?), Self-generated Content (e.g., “when typing a message (on a forum or social network) about coronavirus or related topics, how do you express your opinion, thoughts or feelings in writing”?), Reliability (e.g., “when you search the internet for information on coronavirus or related topics, how easy or difficult is it for you to check different websites to see whether they provide the same”?), and Determining Relevance (when you search the Internet for information on the coronavirus or related topics, how easy or difficult is it applicable to you?). The reliability coefficient values of the four subscales were acceptable to good (0.70 < *α* < 0.83) (59).

### Procedure

This survey procedure was officially endorsed by the University of Education, Winneba's Ethical Review Board (ERB) with a reference number DAA/P.1/Vol.1/39. Further approval was sought from headmasters of all secondary schools in the Northern region who took part in the study. Regardless of one's tribe, every secondary school student who had attended school and lived in any part of the Northern regions for more than 10 years and could read fluently, comprehend and write in the English language was eligible to be involved in the study. Twelve (12) research assistants were trained and taken through the survey instrument from beginning to end with each instruction and item thoroughly explained to them to help with data collection after seeking their voluntary consent to assist in the data collection process. The researchers then began the recruitment process by visiting the secondary schools involved with the research assistants to establish a good rapport and discuss the rationale of the study with both teachers and students. Additionally, each item on the survey instrument was discussed and explained in detail to all students during which there was an opportunity for further clarifications on any item if need be.

Prior to collecting the data, all the participants were asked to sign written informed consent forms to declare their readiness and willingness to be involved in the study. Participants were also told that involvement in the study was purely voluntary and that they had the liberty to continue responding to the items or withdraw at will. Moreover, they were assured of the confidentiality of the responses they would provide. Anonymity was also ensured by asking participants not to provide their names on the survey instruments. All COVID-19 safety protocols were adhered to by providing nose masks and hand sanitizers to each participant. Water, liquid soap and tissue papers were also provided to ensure that the process did not expose any participant to the risk of COVID-19 infection.

Following adherence to all ethical considerations, the DHL survey instruments were distributed to the participants in their various classrooms during their free periods with the help of the research assistants to respond to the survey items. Translation of items on the survey instrument was deemed unnecessary because participants could all read fluently and comprehend the English language. The content of the instrument was explained to the study participants. Responding to the survey items took about 15–20 minutes after which all answered questionnaires were retrieved and sealed in brown envelopes for safe keeping. The entire data collection process lasted for approximately two (2) months.

### Statistical analyses

The data were first screened for missing data; however, none was found. Primarily, the data were analyzed using EFA and CFA to address the objectives of the research. Both the EFA and CFA were performed based on the polychoric approach to factor analysis. Before the major analyses, the data were screened for data entry errors, outliers and any abnormalities. Descriptive statistics and item analyses were performed by exploring the association between the items (polychoric correlation), median, skewness and kurtosis of the items. Statistics on item location and adequacy indicators were also assessed to decide whether some items were adequate to be used in the EFA. Three indices were considered (i.e., Quartile of Ipsative Means (QIM), Relative Difficulty Index (RDI), and Measure of Sampling Adequacy (MSA) based on the recommendations of Lorenzo-Seva and Ferrando (60). Regarding QIM, in a normal range test, a few of the item estimates should be found in the extreme positions in the quartiles and the majority should be found in the middle quartile. For RDI, which evaluates the position of the items, nearly 75% of item values should fall between 0.40 and 0.60, and for MSA, items with estimates below 0.50 should be removed as they measure the same domain as the rest of the items.

The polychoric correlation matrix was performed based on the Bayes Modal Estimation using Monte Carlo simulation (61). The EFA was, therefore, conducted using optimal parallel analysis based on minimum rank factor analysis (62). The use of optimal parallel analysis suggests that the focus of the EFA is to identify major factors. The Promin approach (i.e., oblique rotation method) was used for the factor rotation. The EFA was performed using the FACTOR computer programming software (Version 12.1) (63). This research proposed 4-factor model which was strictly first-order and this is because there was no evidence from previous studies that the DHLI lends itself to the second-order model (36–39). The CFA was further conducted in the R-studio environment using the lavaan package (64) with the diagonally-weighted least squares (DWLS) approach to estimation. With the sample size of more than 400 cases, the DWLS was found appropriate although the weighted least squares- mean and variance (WLSMV) could also equally provided better fit indices (65). The following indices were used to judge the model fit of the specified models: Standardized Root Mean Square Residual (SRMR < 0.08), Goodness-of-Fit Index (GFI,  > 0.90), Root Mean Square Error of Approximation (RMSEA < 0.10), Comparative Fit Index (CFI > 0.90) and Tucker-Lewis Index (TLI > 0.90) (66). As a preliminary analysis, the covariance error matrix was inspected and it came out that there is no covariance error structure.

The ordinal alpha reliability estimate was used for judging the reliability of the DHL dimensions. This reliability estimation approach which was proposed by Zumbo et al. (67) has been found to accurately estimate the reliability coefficient compared to Cronbach's alpha. The ordinal alpha uses the polychoric correlation matrix for estimating the reliability coefficient involving ordinal data (68). The confidence intervals for the reliability estimates were also computed to guide the interpretations. A measurement invariance test was also conducted using the multiple indicators of the distinct models based on gender. The diverse gender group was removed from the measurement invariance analysis because they were quite small and this might have affected the results of the invariance test.

## Results

### Item analysis statistics

The descriptive statistics for the items, which include the item-correlation, median, skewness and kurtosis among others, are presented in Table 1.

**Table 1:** Polychoric matrix, median, skewness and kurtosis of the items.

Domain	Search			Express			Evaluate			Relevance		
Items	1	2	3	4	5	6	7	8	9	10	11	12
1	1.00											
2	0.40	1.00										
3	0.44	0.56	1.00									
4	0.52	0.37	0.37	1.00								
5	0.43	0.58	0.56	0.49	1.00							
6	0.44	0.53	0.55	0.40	0.62	1.00						
7	0.54	0.45	0.38	0.57	0.47	0.45	1.00					
8	0.47	0.48	0.50	0.46	0.57	0.56	0.56	1.00				
9	0.39	0.49	0.54	0.34	0.57	0.57	0.41	0.53	1.00			
10	0.20	0.25	0.24	0.23	0.20	0.27	0.17	0.16	0.16	1.00		
11	0.10	0.14	0.15	0.07	0.17	0.14	0.03	0.12	0.17	0.41	1.00	
12	0.10	0.12	0.12	0.02	0.12	0.13	0.06	0.05	0.15	0.31	0.49	1.00
Median	2.43	2.51	2.56	2.40	2.53	2.51	2.37	2.59	2.70	1.99	2.07	2.24
Skew	0.11	0.08	−0.06	0.166	0.02	0.02	0.17	0.052	−0.26	0.36	0.24	0.10
Kurt	−1.48	−1.33	−1.39	−1.42	−1.26	−1.43	−1.41	−1.26	−1.34	−1.20	−1.13	−1.31
QIM	3	3	3	3	3	3	3	3	3	1	2	2
RDI	0.61	0.63	0.64	0.60	0.63	0.63	0.59	0.65	0.67	0.50	0.52	0.56
MSA	0.92	0.93	0.93	0.89	0.92	0.93	0.89	0.93	0.93	0.79	0.68	0.66

The associations among items under the same factor were found to be fairly moderate (69). For example, the “search” construct domain yielded correlation coefficients between 0.40 to 0.57. Similarly, the “express” sub-scale had coefficients ranging between 0.40 to 0.62. The median values ranged from 1.99 to 2.59 (see Table 1). Whereas the skewness values ranged from 0.06 to 0.36, the values for the kurtosis estimate were between −1.48 to −1.20. Both the skewness and kurtosis values were within acceptable limits (70). Furthermore, the QIM, RDI and MSA values showed that all the items were appropriate and sufficient for the EFA (60). Although the correlation coefficients for some of the items were low ( < 0.20), these items were maintained for two reasons. First, the sample size for the analyses was selected to take into consideration the low correlation coefficients. Secondly, simulation studies (71) have advised against removing items based on low correlations when those items have sufficient factor loadings in a CFA analysis.

### Adequacy of polychoric correlation matrix and model fit

The determinant of the polychoric correlation matrix was found to be appropriate, with a value of 0.008. The Kaiser-Meyer-Olkin (KMO) test also showed a good estimate of 0.898 which was greater than 0.60 (72). Further, Bartlett's statistic showed a significant test result, *χ*2 (66) = 6741.0, *p* < 0.001, reflecting that the EFA was appropriate (69). The RMSR was less than 0.08 (SRMR = 0.019) showing model adequacy.

### Factor solution of DHLI

With the data satisfying the adequacy assumptions, the EFA was performed to assess the number of factors of the DHLI. The outcome of the optimal parallel analysis is presented in Table 2.

**Table 2:** Output from optimal parallel analysis based on Minimum rank factor analysis.

Variable	Real-data % of variance	Mean of random % of variance	Variance percent	Cumulative common variance%	Factor determinacy index
1	47.1214^a^	16.9402	37.26	37.26	0.862
2	15.9443^a^	15.1695	12.61	49.87	0.952
3	13.6344^a^	13.5382	10.78	60.65	0.901
4	11.9833^a^	11.9727	9.48	70.13	0.939
5	9.5304	10.4158			
6	8.1841	8.9546			
7	7.0064	7.5178			
8	5.6477	6.0790			
9	3.1033	4.6335			
10	2.6344	3.1046			
11	1.6630	1.6741			

^a^ Number of factors retained.

The results from the optimal parallel analysis revealed a four-factor structure of the DHLI (see Table 2). The analysis further showed that the four factors accounted for about 70.13% of the variances in DHL of secondary school students. The factor determinacy index was also found to be adequate with all the factors having an estimate greater than 0.80.

### Confirmatory factor analysis

The construct validity of the DHLI was assessed using the following indicators: factor loadings, average variance extracted (AVE), and reliability coefficient from the CFA model.

### Model fit

The model fit of the specified model (4-factor structure, 12-items) was acceptable. The results showed the following model fit for the CFA: *χ*2 (48) = 375.168 (*p <* 0.05), GFI = 0.986, CFI = 0.982, TLI = 0.976, SRMR = 0.057, and RMSEA = 0.070. Except for the chi-square indicator, all the others had adequate model fit indices.

### Construct validity

The details of the analysis are presented in Table 3.

**Table 3:** Factor loading, AVE, and reliability coefficient.

Label	Dimension	Factor loading	Std. Err	AVE	Ordinal alpha (LLCI, ULCI)	Omega *ω* (LLCI, ULCI)
SE=∼	**Information searching** *When you search the Internet for information on coronavirus or related topics, how easy or difficult is it for you to* …	–	–	0.470	0.743 (0.712, 0.774)	0.756 (0.724, 0.787)
SE1	Make a choice from all the information you find?	0.643*	0.020			
SE2	Use the proper words or search query to find the information you are looking for?	0.701*	0.017			
SE3	Find the exact information you are looking for?	0.711*	0.017			
EX =∼	**Self-generated content** *When typing a message (on a forum, social network) about coronavirus or related topics, how easy or difficult is it for you to …*	–	–	0.518	0.906 (0.879, 0.934)	0.799 (0.772, 0.826)
EX1	Clearly formulate your question or health-related worry?	0.624*	0.020			
EX2	Express your opinion, thoughts, or feelings in writing?	0.778*	0.014			
EX3	Write your messages as such, for people to understand exactly what you mean?	0.748*	0.014			
EV=∼	**Reliability** *When you search the Internet for information on coronavirus or related topics, how easy or difficult is it for you to …*	–	–	0.524	0.901 (0.873, 0.929)	0.782 (0.754, 0.811)
EV1	Decide whether the information is reliable or not?	0.729*	0.018			
EV2	Decide whether the information is written with commercial interests (e.g., by people trying to sell a product)?	0.741*	0.015			
EV3	Check different websites to see whether they provide the same?	0.702*	0.017			
DR=∼	**Determining Relevance** When you search the Internet for information on the coronavirus or related topics, how easy or difficult is it for you to…	–	–	0.582	0.881 (0.848, 0.914)	0.720 (0.686, 0.754)
DR1	Decide if the information you found is applicable to you?	0.743*	0.030			
DR2	Apply the information you found in your daily life?	0.718*	0.030			
DR3	Use the information you found to make decisions about your health (eg, on protective measures, hygiene regulations, transmission routes, risks and their prevention)?	0.824*	0.027			

LLCI, Lower limit confidence interval; ULCI, Upper limit confidence interval.

*loadings significant at *p* <0.001.

The outcome of the analysis in Table 3 showed that, for the information sharing domain, the factor loadings ranged from 0.643 to 0.711, with an AVE of 0.470 and ordinal alpha reliability of 0.743, *CI*(0.712, 0.774). The AVE value for the information searching domain failed to meet the recommended cut-off of > 0.5; this could be due to low item variances in explaining the construct as well as the number of items. For the self-generated content sub-scale, the loadings were from 0.624 to 0.778, with an AVE of 0.518 and ordinal alpha reliability of 0.906, *CI*(0.879, 0.934). Similarly, the reliability domain, as well as the determining relevance dimension, also had items with factor loadings of 0.702 to 0.741 and 0.718 to 0.824, respectively. The reliability estimate based on the omega *ω* reliability procedure showed coefficients ranging between 0.720 to 0.799. These two dimensions also had AVE values greater than 0.50 and reliability estimates higher than 0.70 (73). For all the items, the loadings were sufficient (74). Except for the “information searching” dimension, the other dimensions showed a sufficient level of AVE indicating an adequate level of construct validity (75).

### Inter-factorial correlations

The inter-factorial correlation is presented in Table 4.

**Table 4:** Inter-factorial correlations.

	Information searching	Self-generated content	Reliability
Information searching	1		
Self-generated content	0.68	1	
Reliability	0.45	0.42	1
Determining relevance	0.52	0.54	0.55

**Figure 1:**
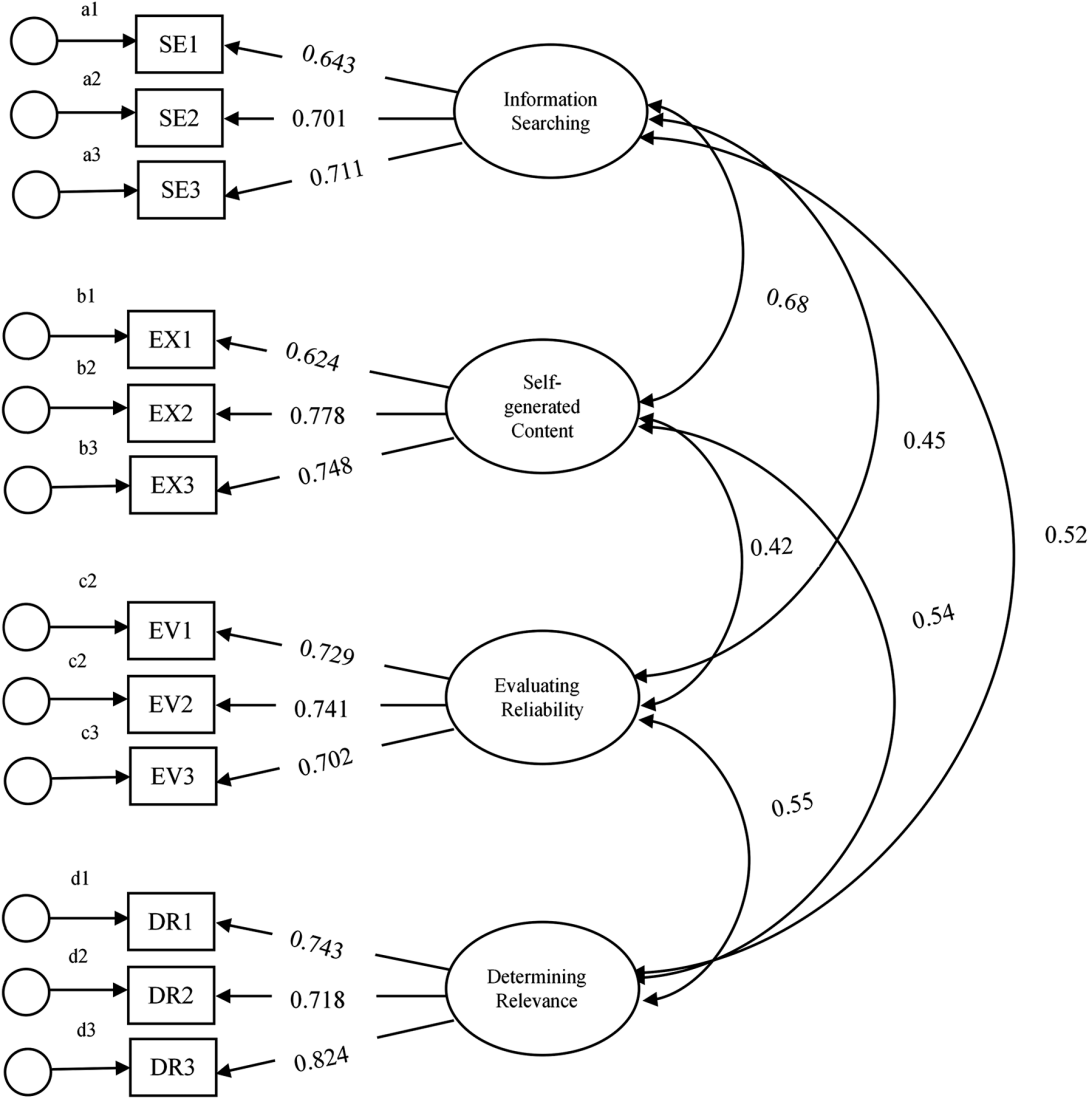
First order CFA model with 4-factor structure and 12 items.

As presented in Table 4, the correlation coefficients ranged between 0.68 and 0.42. For instance, the relationship between self-generated content and information searching was 0.68 whereas the relation between reliability and self-generated content is 0.42 (also see Figure 1).

### Measurement invariance for gender

The study tested for measurement invariance for gender (see Table 5).

**Table 5:** Multiple indicators for measurement invariance for gender.

Indicators	Male	Female	Difference
Chi-square	9711.13*	9716.16*	5.03
Comparative Fit Indices (CFI)	0.982	0.985	0.003
Tucker-Lewis Index (TLI)	0.974	0.975	0.001
Goodness of Fit (GFI)	0.989	0.984	0.005
Root mean square error of approximation (RMSEA)	0.049	0.043	0.006
Standardized root mean square residual (SRMR)	0.054	0.056	0.002
McDonald Fit Indices (MFI)	0.980	0.981	0.001

*loadings significant at *p* <0.001.

Two distinct CFA models were fitted for male (*χ*2 = 9711.13, *p* < 0.001; CFI = 0.982; TLI = 0.974; GFI = 0.989; RMSEA = 0.049; SRMR = 0.054; MFI = 0.980) and female (*χ*2 = 9716.13, *p* < 0.001; CFI = 0.985; TLI = 0.975; GFI = 0.984; RMSEA = 0.043; SRMR = 0.056; MFI = 0.981). The measurement invariance was tested based on the recommendations of Chen (76) (i.e., achieving -0.01 change in CFI,  ≤ 0.015 change in RMSEA and SRMR change cut-off of 0.015 (residual/scalar invariance) or 0.030 (metric invariance). Following the suggestion of Chen (76), the measurement invariance of the DHLI was established with *Δ*CFI of 0.003, *Δ*RMSEA of 0.006 and *Δ*SRMR of 0.002. The results further showed that scalar/residual and metric invariance were satisfied with the use of the instrument.

## Discussion

In this study, the validity of the DHLI was assessed among secondary school students in Ghana, specifically, by examining the factor solution and construct validity through the polychoric factor analytic procedure. The study revealed that, within each dimension, the items were moderately related confirming observations from previous studies (36, 38). However, the inter-dimension item relationships were fairly low signifying homogeneity among the items based on the sub-constructs they measure. There was enough evidence of model adequacy for the polychoric analysis. A 4-factor solution was derived based on the optimal parallel analysis. The 4-factor solution explained more than two-thirds (70.13%) of DHL of secondary school students. Further, a very strong relation was found between the true DHL of secondary school students and that which was estimated. This finding confirms that of previous studies (14, 22, 27, 35–37). Martins et al. (36), for example, found that the 4-factor solution explained 59.5% of the variance in the DHL inventory among university students in Portugal. The study of Rivadeneira et al. (37), though confirming the current study, it rather identified a 5-five factor solution. This reflects all the four dimensions in the current validation, and in addition, the privacy protection dimension which was not considered in the current study. Additionally, the four dimensions are reflected in the seven dimensions originally identified by van der Vaart and Drossaert (27).

The 4-factor solution was confirmed in a CFA with acceptable model fit indices. Additionally, all the item loadings for the various sub-dimensions were greater than 0.70, except items SE1 (…make a choice from all the information you find?) and EX1 (…clearly formulate your question or health-related worry?) which had 0.643 and 0.624 loadings, respectively. Even with these items, they explained about 40% of the variance of their latent traits. Overall, the item loadings were adequate in the measurement of the sub-dimensions of DHL among secondary school students in Ghana. Except for the information searching dimension, which had an AVE of 0.47, all the other dimensions had AVEs greater than 0.50, and these met the minimum recommended threshold (74, 75). Notably, in the case of the information searching dimension, even though the AVE was not up to the recommended level, it was very close to 0.50 (i.e., an AVE value of 0.47), and for that matter considered adequate considering the number of items under the dimension (75). The low AVE of the information searching dimension also might be due to the notion that the errors of measurement are larger than the variances explained by the information searching construct and the accuracy of the item contributions as well as the information searching construct. Nevertheless, the reliability coefficient of 0.743 for the information searching dimension provided evidence of convergent validity and thus, the low AVE value for the dimension might not be a concern as suggested by Fornell and Larcker (75). Furthermore, the other dimensions of the DHLI also had sufficient internal consistencies. The outcome of this study is in line with previous studies in other countries that have reported high internal consistencies and AVEs (36–39). It must be noted that not all consistencies functioned the same for secondary school students, as different dimensions have varying levels of precision in measuring the various aspects of DHL.

The study further established measurement invariance of the DHLI based on gender. This result implies that the DHL construct had a similar meaning and structure for both male and female students. Consequently, the DHL construct can be meaningfully construed across both male and female students, justifying the utilisation of the instrument for scaling students into their DHL levels irrespective of their gender.

The DHLI is functionally applicable in other contexts such as in Ghana. More importantly, the current study provides diverse applicability in terms of the cohort of students. While the previous studies used university students who are early adults aged between 20 and 24 years, participants in the current study used secondary school students who were averagely aged 18 years, thus, late adolescents. Generally, it can be said that the DHLI is ecologically robust and age-wise reproducible, though more studies are needed to further validate it. Considering the outbreak of diseases and the proliferation of technology and other ICT devices, the development and re-validation of DHLI is timely as it provides useful information on the easiness or otherwise difficulty in accessing, evaluating, and use of health-related information through the blend of technology and competence among students.

### Limitations and future directions

Although this research provides direction to future studies on the psychometrics of the DHLI, it has some limitations. The study involved secondary school students clustered in the Northern zone of Ghana. These students may possess some characteristics which are different from students from other regions. Furthermore, the findings of this study on gender measurement invariance differed from what exists in literature and this presents mixed research findings. Further studies should consider pursuing the issue of gender measurement invariance since gender is likely to be a key variable in the measurement of DHL.

### Implications for educational and health practice

The study offers much insight into the adaptation and utility of the DHLI among the youth within the Ghanaian setting. Educational and public health practitioners could make use of the DHLI to identify students with inadequate DHL for appropriate interventions to be rolled out to them. This is particularly important since recent studies have established a relationship between DHL and protective health behaviours (11, 14, 15). With the acceptable applicability of the DHLI, educators could adapt the instrument to study the sense of digital literacy in a general search for educational materials for learning by students. The DHLI can offer a platform where the efficacy of intervention digital health programs can be tested.

## Conclusion

The findings from the study support the validity of the DHLI and consequently, its utility within the Ghanaian context. With the growing need for digital health literacy among younger people globally, the DHLI provides sufficient grounds for scaling them based on their level of literacy. There is a need for the instrument to be adapted and re-validated in Ghana and among the different samples to widen its reproducibility. The study establishes that secondary school students digital health literacy can be understood from four perspectives (i.e., searching for information, self-generated context, assessing the reliability of the information and determining the relevance of the information). Although the findings of the study are useful to start the discussions on the utility of DHLI, a number of challenges were identified and other areas of validation could not be covered. For instance, the information searching dimension needs further investigation on why the AVE estimate failed to reach the recommended level. Also, it appears the inter-factorial correlation between the “information searching” and “self-generated content” was relatively high and this could have implications on the further investigation of the latent structure of the instrument. The study recommends that future studies should conduct discriminant analyses and differential item analyses to understand the study's result and other features of the instrument.

## Data Availability

The raw data supporting the conclusions of this article will be made available by the authors, without undue reservation.
